# Intergenerational Programming During the Pandemic: Transformation During (Constantly) Changing Times

**DOI:** 10.1093/geroni/igab046.1553

**Published:** 2021-12-17

**Authors:** Shannon Jarrott, Skye Leedahl, Donna Butts

## Abstract

Implementing intergenerational programming amidst the COVID-19 pandemic has required creativity, partnership, and dedication to the work. Most intergenerational programs involving in-person meetings or events are accompanied by guidelines to protect participant health and safety. Programming is routinely cancelled or postponed due to poor weather or contagious illness, particularly when a vulnerable population is involved. The needs for safety precautions and continued intergenerational contact were both amplified during the pandemic, leading many to modify or innovate ways to engage generations rather than eliminate contact for extended periods. Technology has afforded new approaches to engage young people and older people with each other; non-technological ways have also proven effective. This symposium will address strategies used to implement intergenerational programs during the pandemic. Authors will highlight lessons learned and strategies they expect to retain in the future. The first paper describes a pivot in nutrition programming designed for a shared site with preschool children and frail older adults. In paper two, authors discuss their partnership-based approach shifting to remote offerings of Cyber-Seniors programming. Paper three addresses how MentorUp Service-Learning expanded its reach by adaptations to virtual programming for older adults in retirement communities. The final paper presents evaluation data comparing arts programming delivered in-person pre-pandemic and again virtually during the pandemic. In each case, researchers and community partners learned techniques to maintain their programmatic foci. Some projects developed strategies they plan to maintain post-pandemic. Donna Butts, Executive Director of Generations United serves as the symposium discussant.

